# Association of Lipoprotein Lipase (*LPL*) Variants rs8176337, rs303, and rs304 with Body Mass Index and Total Cholesterol

**DOI:** 10.3390/ijms26157282

**Published:** 2025-07-28

**Authors:** Suzanne A. Al-Bustan, Ahmad E. Al-Serri, Amani M. Al-Adsani, Lavina Miranda, Babitha G. Annice, Hala Hamdan, Majed A. Alnaqeeb

**Affiliations:** 1 Department of Biological Sciences, Faculty of Science, Kuwait University, P.O. Box 5069, Safat, Kuwait City 13060, Kuwait; amani.aladsani@ku.edu.kw (A.M.A.-A.); lavina.l.miranda@ku.edu.kw (L.M.); babitha.george@ku.edu.kw (B.G.A.); hala.hamdan@grad.ku.edu.kw (H.H.); m.alnaqeeb@ku.edu.kw (M.A.A.); 2 Unit of Human Genetics, Department of Pathology, Faculty of Medicine, Kuwait University, Kuwait City 13060, Kuwait; ahmad.alserri@ku.edu.kw

**Keywords:** LPL, genetic association, BMI, lipids, sequence variants

## Abstract

Several single-nucleotide polymorphisms (SNPs) across the lipoprotein lipase (*LPL*) gene have been found to be associated with dyslipidemia and obesity. Several InDels and SNPs in exon 1, intron 2, and intron 7 have been reported; however, their association with lipid parameters and body mass index (BMI) remains unclear. Here, we aimed to investigate the relationship among *LPL* variants, lipid levels, and BMI in a Kuwaiti population. Sanger sequencing was performed on three targeted regions of the *LPL* gene. Based on the minor allele frequency, Hardy–Weinberg equilibrium, and linkage disequilibrium, five SNPs were selected and genotyped in a cohort of 688 Kuwaiti samples to investigate their association with lipid levels and BMI. A total of 30 variants (6 InDels and 24 SNPs) were identified; of them, 5 SNPs (rs1800590, rs74377536, rs8176337, rs303, and rs304) were selected for their association with BMI and lipid levels. The G-allele of rs8176337 was found to be associated with increased BMI (β = 1.41; 95% confidence interval = 0.22–2.60; *p* = 0.02). In addition, an association was observed for rs303 and rs304 with both cholesterol and LDL (*p* < 0.05). Overall, our results demonstrate an association between *LPL* variants and lipid levels, and the observed association between rs8176337 and BMI was novel.

## 1. Introduction

The role of intronic sequence variants in modulating gene expression remains unresolved, with many studies reporting conflicting results regarding their association with complex traits [1]. Large structural genes consist of numerous and large introns, wherein hundreds of variants have been identified with minor allele frequencies (MAFs) ranging from <0.01 (rare and very rare) to >0.05 (common). The reported associations of variants with complex traits such as dyslipidemia are inconsistent. Variants identified in the introns and non-coding regions of candidate genes can present challenges in assessing their actual contribution to disease risk in various populations [2]. These include lipoprotein lipase (*LPL*), which is known for its role in modulating triglyceride (TG) levels by catalyzing the hydrolysis of TG to non-esterified free fatty acids and 2-monoacylglycerol. It has also been suggested that LPL regulates the release of fatty acids and its transport through the formation of chylomicrons, very-low-density lipoproteins (VLDLs), and high-density lipoproteins (HDLs) [3,4]. The levels of these lipoproteins are influenced by a variety of lipid metabolism and transport mechanisms, which result in defective LPL structures/functions [5], and may often manifest into dyslipidemia and other metabolic disorders including obesity.

LPL consists of 475 amino acids and with a size of 55 kDa that includes the 27-amino acid signal peptide, which is cleaved to produce a mature protein of 448 residues. The encoding gene spans 30 kb and is localized to the short arm of chromosome 8p22 [6]. The full *LPL* gene consists of 10 exons varying in size. Exons 1–9 have an average size of 105–276 bp while exon 10 is much larger at 1948 bp, which encodes the full 3′ untranslated region (UTR) [6]. The 5′ UTR is encoded by exon 1 and includes the sequence for the signal peptide, as well as the first 2 amino acids of the mature protein [7]. The remaining 446 amino acids are encoded by exons 2–8. Hundreds of variants, including single-nucleotide polymorphisms (SNPs) within both coding and non-coding regions, have been identified and reported in Ensemble. Insertions/deletions (InDels) have been mostly reported in the non-coding regions.

Studies have demonstrated the importance of screening for *LPL* variants to establish an association between plasma lipid levels. Sequence analysis employing Sanger sequencing of the *LPL* gene locus across different populations has revealed novel and common variants associated with an increased risk of developing dyslipidemia and metabolic disorders including obesity [8,9,10,11,12,13]. In a recent study that screened the entire gene with the flanking sequences, 46 novel variants were identified, including 11 InDels [14]. Three InDels were reported to have the highest MAF, albeit considered rare (<0.01). These variants were localized in exon 1, intron 2, and intron 7. However, the roles of these InDels have not yet been established.

Moreover, numerous variants, in both the coding and non-coding regions of the *LPL* gene have been reported to be associated with complex diseases (15–17) that are linked to abnormal lipid profiles. Recently, however, more attention has shifted to non-coding intergenic regions, and many emerging findings have shown a correlation between intronic variants and manifestation of metabolic diseases [1]. Several intergenic regions in *LPLs* may be functionally relevant to various phenotypes and could be associated with metabolic disorders such as diabetes and obesity, as well as coronary heart disease [11,12,13,15,16,17]. An investigation on the genetic association of intronic variants, employing population and targeted gene analysis, would be required to address the gap in the functional role of such variants with fluctuations in lipid levels and BMI [18,19], especially because LPL plays a vital role in modulating TG levels. Accordingly, based on the previous study [14], as mentioned earlier, that reported novel InDels and SNPs in exon 1, intron 2, and intron 7, validation and functional analysis are warranted to determine their association with lipid parameters and BMI. Therefore, this study aimed to investigate the association of variants in exon 1 (non-coding amino acid sequence), intron 2, and intron 7 of the *LPL* gene with Body Mass Index (BMI) and variations in lipid levels including TG, total cholesterol (TC), and low-density lipoprotein (LDL), as well as HDL, which are commonly presented with dyslipidemia in the Kuwaiti population.

## 2. Results

### 2.1. Sequence Analysis and Variant Identification

The sequences generated for the three regions have been deposited in GenBank under the accession number MN180200. The three InDels (exon 1: 5202 Ins/CC, intron 2: 14866_14876 del/An, and intron 7: 25708–25710 del/Tn) were validated (Supplementary Figure S1) and identified with MAF ≤ 0.05. Both rs530320828 and rs71205952 deviated from HWE, making them unsuitable for genetic association analysis (Table 1).

**Figure 1:**
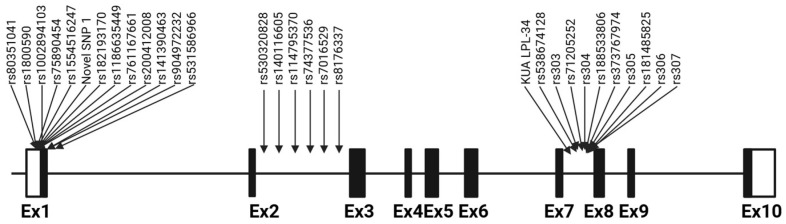
Distribution of the variants identified within exon 1 and introns 2 and 7 of the *LPL* gene.

The advantage of using Sanger sequencing to genotype variants is the identification of other variants in the fully sequenced regions. In this study, 30 variants were identified (Figure 1); they included 6 InDels and 24 SNPs, of which 13 were transitions (Supplementary Table S1). The MAF distribution among the cohort for all the variants ranged across “very rare,” “rare,” and “common” (Supplementary Figure S2). The “very rare” variants were more frequent (70%) in the cohort than the “common”, which included only six variants.

### 2.2. Genotype Distribution and Haplotypes

The genotype and allele frequencies of all 30 variants were estimated using a simple gene-counting method. A summary of the genotype frequency distribution of the 30 identified variants is provided in Supplementary Table S2. The genotype distribution of three of the variants was found to deviate from HWE, including two of the studied InDels (intron 2: 14875–14876 del/A and intron 7: 25708–25710 del/TTT) and rs7016529 T > C. Linkage disequilibrium (LD) was assessed for the six common SNPs (MAF > 5%). A strong LD (r^2^ = 0.99) was observed between rs304 and rs305 (Supplementary Figure S3). Haplotype analysis showed that rs303, rs304, and rs305 fell under the same haplotype block with the construction of three haplotypes, the most common one being GTA (frequency 78.4%).

Analyzing all 24 SNPs in the *LPL* gene in the cohort showed the construction of the same haplotype block, with the highest significant LD found between rs304 and rs305 (Supplementary Figure S3).

### 2.3. Genetic Association

Based on the HWE, MAF, and haplotype analyses, five SNPs (rs1800590, rs74377536, rs8176337, rs303, and rs304) were selected to assess their relationship with BMI and lipid levels. The genotypic distributions of the five variants are provided in Supplementary Tables S3 and S4.

Using analysis of variance, we compared the means of our selected SNPs with lipid levels and BMI (Table 2). A significant association was found between the G-minor allele of rs8176337 and increased BMI (*p* = 0.036; Table 2). No association was observed between any of the remaining SNPs and BMI (Table 2). In addition, the C-minor allele of rs303 was associated with increased TC and LDL levels (*p* = 0.023 and *p* = 0.036, respectively; Table 2). Moreover, the G-minor allele of rs304 was also associated with both TC (*p* = 0.034) and LDL levels (*p* = 0.034) (Table 2). No association was observed between the remaining SNPs and lipid levels (*p* > 0.05).

The significant SNPs were further assessed using linear regression and adjusted for age, sex, and TG. The G-allele of rs8176337 remained significantly associated with BMI after adjusting for confounders (β = 1.41; 95%CI = 0.22–2.60; *p* = 0.020) (Table 3). Both age and TG were significantly (*p* < 0.001) associated with an increase in BMI by 0.13 and 2.32, respectively, with TG identified as a stronger predictor.

Similarly, the relationship across the selected significant SNPs, adjusted for both age and sex, was assessed using linear regression (Table 4). The G-allele of rs303 remained significantly associated with both TC (β = 0.210; 95% CI 0.034–0.386; *p* = 0.020) and LDL (β = 0.195; 95%CI 0.039–0.351; *p* = 0.014) after adjusting for age and sex (Table 4A). In addition, age, but not sex, was found to predict TC and LDL levels (*p* < 0.001). Likewise, linear regression analysis showed the G-allele of rs304 to remain associated with increased TC (β = 0.174; 95%CI 0.034–0.314; *p* = 0.015) after adjusting for both age and sex (Table 4B). The G-allele was also found to be associated with LDL (β = 0.174; 95% CI 0.050–0.298; *p* = 0.006).

## 3. Discussion

The current study describes the sequence variants of selected non-coding regions that might play a role in regulating *LPL* gene expression and/or splicing. The rationale behind the study is based on previous reports [8,9,10,11,12,13] that described the importance of investigating non-coding variants in the *LPL* gene locus for their possible association with metabolic disorders. In this study, all genetic variants identified in the targeted sequences of exon 1, introns 2 and 7, and their genotypes were identified using Sanger sequencing. Variants with an MAF greater than 5% that did not deviate from HWE and that met other inclusion criteria (based on LD and haplotype analysis) were further investigated for their association with increased lipid levels and BMI, which are presented with high prevalence in Kuwait. Different studies have reported sequence variants of the *LPL* gene locus across different populations, revealing novel and common variants associated with an increased risk of developing dyslipidemia and BMI [8,9,10,11,12,13]. The findings in this study support the importance of non-coding variants and their possible role in modulating LPL activity.

The study identified 30 variants in partial regions of exon 1 (5′UTR), intron 2, and intron 7. Five variants, mainly SNPs, were further analyzed for their genetic associations with lipid levels and BMI. An association between variant rs8176337 in intron 2 and BMI was observed in the cohort of Kuwaiti samples, whereas intron 7 variants rs303 and rs304 were found to be associated with increased TC and LDL-C levels. Our findings on the genetic association of *LPL* variants with lipid levels are consistent with those of several previous studies [8,9,14,18,20]. Our results are consistent with reported association between intronic variants and TC levels that have been reported in several studies [8,14,20], including the association of a novel rare variant in intron 3 with HDL-C [14], a common variant rs252 in intron 4 with HDL-C [8], variants rs34123038 and rs74304285 in intron 2 with opposing effects on TG and HDL-C [21], intron 6 rs283 with HDL [22], and rs295 with LDL [23]. In addition, two SNPs near the *LPL* gene and rs10096633 have been shown to be associated with HDL-C [21]. However, studies on the association between *LPL* variants and BMI are still limited [13,21,24]. In a study conducted on the Asian populations, the promoter variant rs1800590 was reported to be associated with obesity suggesting a population specific role of the variant with obesity. This aligns with our findings, wherein a novel association of rs8176337 with obesity was found in the Kuwaiti population, which is mostly of Arab ancestry.

LPL is well documented as a modulator of TG, and common variants of the *LPL* gene are known to influence TG levels [17] through tissue-specific alterations in gene expression. Goodarzi et al. [25] demonstrated that LPL is localized to the capillary endothelium of adipose tissue, where it is the most abundant. Moreover, the contribution of LPL to adiposity and lipid metabolism has been demonstrated to influence energy storage and utilization. Specific metabolic demands, such as an increase in energy intake, might shift the expression from one tissue to another, leading to decreased TG hydrolysis [26] and increased weight, which is reflected by the increased BMI observed in this study. The results indicated a strong predictive effect of rs8176337 on increased BMI, independent of TG, which could possibly be the outcome of altered gene expression. Human splice finder [27] was used to predict the functional consequences of rs8176337. The variant could activate an intronic cryptic donor site, leading to splicing that possibly involved intron retention or exon skipping (Supplementary Table S4). In a recent review [1] on the potential role of intronic variants, possible mechanisms that could explain the role of rs8176337 in altering *LPL* gene expression were suggested. The rate of transcription is mediated by the 5′ splice sites (SS), which could be altered by various splicing factors. Intronic variants have also been suggested to influence the efficiency of mRNA translation [1,24], thereby affecting the catalytic activity of LPL and TG hydrolysis; however, the underlying mechanisms remain unresolved. One possible scenario is that the altered splicing in the intron can lead to a change in the N-terminus of the amino acid sequence (residues 54–64) required for the formation of the β5 loop [26], crucial for LPL activity, which is encoded by the flanking coding regions of exons 1 and 2. In this study, no association between the studied variants and TG levels was observed. The lack of association may be the result of the TG levels being measured after a 12–14 h fasting where postprandial TG levels would reflect a more accurate function of LPL. In addition, non-coding variants would have an indirect effect on LPL activity by either influencing regulatory mechanisms as explained earlier, or by interacting with other gene variants in addition to the possible influence of environmental factors. Moreover, for the clearance of TG, other enzymes such as peripheral lipases may compensate for LPL’s reduced activity associated with the non-coding variants.

Similarly, the functional role of the variants rs303 and rs304 as well as their effect on TC and LDL-C levels observed in this study could be explained by the mechanisms of gene regulation and splicing, although the human splice finder predicted no effect on splicing for these two variants, except for an alteration in the intronic exon splicing silencer (ESS) (Supplementary Table S4). A possible functional role of rs303 and rs304 is suggested by their proximity to rs301. The latter has strong linkage disequilibrium (LD) with the intron 8 variant rs320, which has been established to affect the binding affinity of transcription factors with the TATA promoter, thereby affecting the transcription rate of *LPL* [28]. The variant rs301 has also been suggested to be in LD with other variants, and can, through long-range interactions, act as an enhancer or silencer of splicing that may subsequently affect the expression levels of LPL. As both rs303 and rs304 are near rs301, but not in LD, their functional roles can be attributed to those suggested for rs301. The two variants may have an independent role in modulating the expression levels of *LPL* and may also be interacting with other variants (TG-modulating genes) to produce abnormal lipid levels. Altered LPL levels and/or activity can lead to fluctuations in TC and LDL-C levels via different mechanisms. LPL plays an important role in maintaining intracellular homeostasis of lipoproteins, and HDL-C metabolism is important for the transfer of surface lipids to small HDL particles following lipolysis. That is, LPL influences HDL-C metabolism by hydrolyzing TG-rich lipoproteins, which releases surface lipids and apolipoproteins. These are transferred to small HDL particles, promoting HDL maturation. This process is further supported by LPL’s role in anchoring and bridging to facilitate lipoprotein remnant uptake by interacting with surface receptors on cells including the LDL and VLDL receptors [29]. Furthermore, Heid et al. [16] reported that the association of *LPL* intronic variants with HDL-C levels may be due to alteration or repression of the miRNA-binding affinity on the 3’UTR sites on mRNA for lipid-regulating proteins. The effect of variants in intron 7 on the enzyme structure, and thereby its activity, can be explained by the role of the C-terminal domain (amino acid residues 313–352 in exon 7 and 353–413 in exon 8) in the uptake of lipoproteins by cell surface receptors mediated by LPL as explained earlier [19,29]. Altered binding affinity may lead to fluctuations in lipid levels owing to poor transport, resulting in significant association with lipid levels. Studies have suggested that in response to diet and environmental factors, cell type-specific expression of LPL is often modified, providing a plausible explanation for the association of *LPL* variants with high TC and obesity, which is highly prevalent in the Kuwaiti population due to a fat-rich diet and lifestyle [3,5,14,20]. Furthermore, *LPL* non-coding variants may interact with other variants in genes related to lipid metabolism such as hepatic lipase and apolipoproteins C2 and C3, which are regulators of LPL to reduce LPL activity, thereby affecting TC and LDL-C levels, as well as indirectly increasing BMI. This study had some limitations mainly related to the sample size and findings of the haplotype analysis. Another limitation is the lack of LPL levels that would provide information on the potential effect of the studied non-coding variants on LPL activity.

## 4. Materials and Methods

### 4.1. Sample Description

The collection of samples in this study was approved by both the Joint Committee for the Protection of Human Subjects in Research, Health Sciences Center, Kuwait University and Kuwait Institute for Medical Specializations and by the Kuwait Ministry of Health Ethics Committee and conducted in agreement with the Helsinki guidelines of 1975. Overall, 715 volunteers were recruited from the general Kuwaiti population during their routine visits to the Kuwaiti health centers and hospitals. Written informed consent was obtained from all participants to identify the variants in the selected intronic regions of *LPL*.

None of the selected participants had any metabolic disease at the time of blood collection. Samples from all the volunteers allowed the identification of a wide range of genetic variants. However, only 688 participants (414 females and 274 males) met the inclusion criteria and were considered for the genetic association study cohort. The inclusion criteria were age > 18 years, presence of a documented lipid profile, and BMI. Plasma lipid levels were determined at various clinical laboratories in Kuwait using automated chemistry analyzers (Beckman Unicel DxC 800; Beckman Corporation, Brea, CA, USA) calibrated to the same standards as commercially available reagents, as described previously by Al-Bustan et al. [18]. Total TC, TG, HDL-C, and LDL-C levels were expressed in mmol/L. The reference values for the lipid profiles were based on those set by the Kuwait Ministry of Health as follows: TC = 3.0–5.17 mmol/L, TG = 0.40–1.7 mmol/L, HDL-C = 0.91–2.5 mmol/L, and LDL-C = 1.8–3.2 mmol/L. Table 5 summarizes the demographics of the sample.

### 4.2. DNA Extraction

The salting out method, as described by Miller et al. [30], was used to extract total genomic DNA from all the blood samples. All DNA samples were analyzed to determine their quantity by NanoDrop spectrophotometry and diluted to yield a final concentration of 25 ng/µL for sequencing.

### 4.3. Amplification and Sequencing of the Target Region

The complete published human reference sequence in GenBank (MN180200) was used to design three different primer sets flanking 6680 bp, 449 bp, and 550 bp regions throughout the *LPL* gene in exon 1 (5′ UTR), as well as introns 2 and 7, respectively [14]—spanning nucleotide positions 5182–5458 in exon 1, 14943–15441 in intron 2, and 25565–26114 in intron 7—were designed. PCR was performed with the primers designed, according to Al-Bustan et al. [14], to amplify the *LPL* gene target sequences in the Kuwaiti cohort (*n* = 688). The PCR products were purified by Nucleospin^®^ Gel and PCR Clean-up Kit (Macherey-Nagel, Düren, Germany, 740609.250). The forward primer, starting at the 5′ end, for each of the 3 regions was used to sequence the DNA samples with the BigDye™ Terminator v3.1 Cycle Sequencing Kit (Applied Biosystems, Carlsbad, CA, USA, 4337455). BigDye XTerminator Purification kit (Applied Biosystems, 4376486) was then used to denature the double-stranded DNA from the sequenced products. The samples were taken for bidirectional sequencing on an ABI 3130xl genetic analyzer, and the sequence data were analyzed using the ABI DNA Sequencing Analysis Software (version 2.5). For quality assurance of the generated sequence data, the reverse primer was used to sequence DNA from all the samples and compare their sequence alignment with that generated from the forward primer.

### 4.4. Sequence Data Analysis and Variant Identification

Multiple sequence alignments using the Clustal Omega software (https://www.ebi.ac.uk/jdispatcher/msa/clustalo? accessed on 26 July 2025) were used to analyze the generated sequences. The sequences were compared to the GenBank reference sequence (MN180200). They were screened for novel InDels and other variants in the targeted regions. The MAF distribution among the cohort for all the variants ranged from “very rare,” “rare,” to “common.” Functional prediction analysis of the selected variants was performed using a human splicing finder [27].

### 4.5. Linkage Disequilibrium and Haplotype Construction

LD between the selected SNPs that were in HWE and with MAF ≥ 0.05 was analyzed using Haploview (version 4.2). The squared coefficient of correlation (r^2^) was calculated for each SNP pair. A strong LD between two loci is indicated by the r^2^ value of 1 (black color) or greater than 0.8 (dark gray). In contrast, an r^2^ value of 0 (white) or lower than 0.8 (shades of light gray) indicates a linkage equilibrium. In addition, LD was defined using the standard color scheme of D’/LOD, with bright red indicating strong LD (D’ = 1; LOD ≥ 2), shades of pink/red (D’ < 1; LOD ≥ 2) and blue (D’ = 1; LOD < 2) indicating intermediate LD, and white (D’ < 1; LOD < 2) indicating no LD. Haplotype blocks were created according to the criteria of Gabriel et al. [31], which required 95% of informative comparisons to be in strong LD, defined by the confidence bounds of D’. Based on the haplotype analysis, SNPs were selected for genetic association analysis.

### 4.6. Genotype and Statistical Analysis

The genotype and allele frequencies of all 30 variants of the *LPL* locus were determined using a simple gene-counting method. Deviation from the HWE was investigated using GENEPOP software (Version 4.2). Subject profiles, including plasma lipid levels, are expressed as means ± standard deviation (SD) and percentage, where appropriate. SNPs with an MAF ≥ 5% and in HWE were investigated further. One-way ANOVA was used for the statistical comparison of BMI between the genotype groups of the selected SNPs, whereas Kruskal–Wallis ANOVA was used for the statistical comparison of lipids. To assess the influence of confounding factors, significant SNPs were further analyzed using linear regression adjusted for age, sex, and TG, where appropriate, and were represented as beta (B) coefficients and 95% CI. TG, HDL, and VLDL levels were naturally log-transformed to achieve an approximately normal distribution in linear regression analysis. SPSS software (version 23; SPSS Inc., Chicago, IL, USA) was used for all the statistical analysis, to which *p* < 0.05 was set for statistical significance.

## 5. Conclusions

The current study reported an association between the intronic variant rs8176337 of the *LPL* gene locus with obesity, as well as both rs303 and rs304 with TC and LDL. The findings further validated the importance of intronic variants in the etiology of complex traits, such as obesity. Previous studies and our current findings support the notion that variants can directly affect blood lipid levels. Understanding the role of such variants in increasing the risk of developing dyslipidemia and obesity can provide a future means for screening and management. Future studies are needed to investigate the functional role of intronic variants and their effect on gene expression and protein synthesis.

## Figures and Tables

**Table 1:** Description of the three InDels investigated in this study and their minor allele frequencies (MAFs) in the cohort.

LPL Novels	SNP ID Number	Position Within Gene	Consequence	Global MAF	Ancestral Allele	Variant Allele	HWE *p*-Value
KUA LPL-16	rs1554516247	5202 Ins/CC	5′ UTR Variant	NA	0.997	0.003	0.940
KUA LPL-26	rs530320828	14866_14876 del/A_n_	Intron Variant	AA = 0.0407 (*n* = 204)	0.500	0.500	<0.001 *
KUA LPL-36	rs71205952	25708–25710 del/T_n_	Intron Variant	delTTT = 0.01779 (*n* = 206)	0.500	0.500	<0.001 *

* Deviated from HWE at *p* < 0.05.

**Table 2:** Comparison of means between the genotypes of the selected *LPL* variants and the lipid profiles and BMI.

SNP	Variable ^	W/W	*n*	W/M	*n*	M/M	*n*	W/M + M/M	*n*	*p*-Value *
rs1800590	BMI	27.07 ± 6.66	421	28.18 ± 6.85	49	24.45 ± 0.78	2	28.03 ± 6.75	51	0.330
(T‹G)	TC	4.73 ± 0.94	610	4.73 ± 0.87	76	4.80 ± 1.27	2	4.72 ± 0.87	78	0.890
	TG	1.05 ± 0.79	610	1.16 ± 0.82	76	1.12 ± 0.79	2	1.16 ± 0.82	78	0.349
	VLDL	0.44 ± 0.33	610	0.47 ± 0.34	76	0.45 ± 0.33	2	0.48 ± 0.34	78	0.348
	HDL-C	1.14 ± 0.32	610	1.07 ± 0.28	76	1.29 ± 0.13	2	1.08 ± 0.29	78	0.207
	LDL-C	3.12 ± 0.83	610	3.13 ± 0.70	76	3.10 ± 1.41	2	3.13 ± 0.71	78	0.576
rs74377536	BMI	27.33 ± 7.08	342	26.73 ± 7.08	124	27.38 ± 8.09	6	26.76 ± 5.47	130	0.415
(C‹A)	TC	4.75 ± 0.94	503	4.62 ± 0.89	176	5.46 ± 1.45	9	4.67 ± 0.94	185	0.388
	TG	1.07 ± 0.83	503	1.04 ± 0.70	176	1.23 ± 0.65	9	1.06 ± 0.70	185	0.495
	VLDL	0.44 ± 0.35	503	0.42 ± 0.28	176	0.51 ± 0.26	9	0.43 ± 0.28	185	0.550
	HDL-C	1.13 ± 0.30	503	1.11 ± 0.35	176	1.08 ± 0.39	9	1.11 ± 0.35	185	0.191
	LDL-C	3.13 ± 0.81	503	3.04 ± 0.78	176	3.82 ± 1.24	9	3.09 ± 0.83	185	0.435
rs8176337	BMI	26.69 ± 6.09	302	27.92 ± 7.27	145	28.66 ± 9.09	25	28.03 ± 7.54	170	0.036
(C‹G)	TC	4.71 ± 0.97	437	4.73 ± 0.90	218	4.94 ± 0.72	33	4.75 ± 0.88	251	0.494
	TG	1.08 ± 0.85	437	1.03 ± 0.70	218	1.06 ± 0.80	33	1.04 ± 0.70	251	0.430
	VLDL	0.45 ± 0.37	437	0.42 ± 0.28	218	0.45 ± 0.26	33	0.43 ± 0.28	251	0.372
	HDL-C	1.13 ± 0.33	437	1.12 ± 0.29	218	1.10 ± 0.34	33	1.12 ± 0.30	251	0.956
	LDL-C	3.09 ± 0.82	437	3.15 ± 0.83	218	3.24 ± 0.68	33	3.17 ± 0.82	251	0.257
rs303	BMI	27.10 ± 6.56	396	27.40 ± 7.32	71	29.46 ± 6.60	5	27.54 ± 7.25	76	0.601
(G‹C)	TC	4.69 ± 0.93	562	4.88 ± 0.944	118	4.96 ± 1.07	8	4.89 ± 0.95	126	0.023
	TG	1.07 ± 0.82	562	1.08 ± 0.73	118	0.73 ± 0.38	8	1.06 ± 0.72	126	0.838
	VLDL	0.44 ± 0.34	562	0.44 ± 0.29	118	0.31 ± 0.17	8	0.43 ± 0.29	126	0.879
	HDL-C	1.12 ± 0.31	562	1.13 ± 0.31	118	1.20 ± 0.46	8	1.14 ± 0.33	126	0.738
	LDL-C	3.08 ± 0.81	562	3.25 ± 0.84	118	3.41 ± 0.85	8	3.27 ± 0.84	126	0.039
rs304	BMI	26.98 ± 6.46	300	27.17 ± 6.12	147	29.44 ± 10.99	25	27.50± 7.04	172	0.418
(T‹G)	TC	4.66 ± 0.95	428	4.82 ± 0.91	224	4.88 ± 0.82	36	4.83 ± 0.91	260	0.034
	TG	1.09 ± 0.87	428	1.02 ± 0.68	224	1.00 ± 0.53	36	1.02 ± 0.66	260	0.868
	VLDL	0.45 ± 0.36	428	0.41 ± 0.27	224	0.41 ± 0.21	36	0.42 ± 0.27	260	0.806
	HDL-C	1.12 ± 0.32	428	1.14 ± 0.31	224	1.13 ± 0.28	36	1.15 ± 0.31	260	0.195
	LDL-C	3.06 ± 0.80	428	3.21 ± 0.83	224	3.27 ± 0.78	36	3.22 ± 0.83	260	0.034

* ***p*-value for the dominant genetic model**. W/W indicates a major (wild) homozygous genotype, W/M indicates the heterozygous genotype. M/M indicates a minor (mutant) homozygous genotype, W/M+M/M indicates the dominant model; carriers of the minor allele (mutant allele). **^ Units for the variables tested.** BMI: Body Mass Index (kg/m^2^), TC: Total Cholesterol (mmol/L), TG: Triglycerides (mmol/L), VLDL: Very-Low-Density Lipoprotein (mmol/L), HDL-C: High-Density Lipoprotein–Cholesterol (mmol/L), LDL-C: Low-Density Lipoprotein–Cholesterol (mmol/L).

**Table 3:** Linear regression analysis illustrating the association between rs8176337 under a dominant genetic model and BMI after adjusting for age, sex, and TG.

Variable	β-Coefficient	95% CI	*p*-Value
rs8176337	1.41	0.22–2.60	0.020
Age (Female)	0.13	0.04–0.14	<0.001
Sex	−0.48	−1.7–0.73	0.438
TG	2.32	1.40–3.25	<0.001

TG: Triglycerides (mmol/L).

**Table 4:** Linear regression analysis assessing the association of rs303 and rs304 with TC and LDL after adjusting for age and sex under a dominant genetic model.

**A. rs303**				
**Lipid**	**Variable**	**β-Coefficient**	**95% CI**	***p*** **-** **V** **alue**
TC	rs303	0.210	0.034–0.386	0.020
	Age	0.017	0.012–0.022	<0.001
	Sex (Female)	0.001	−0.139–0.140	0.992
LDL-C	rs303	0.195	0.039–0.351	0.014
	Age	0.010	0.006–0.014	<0.001
	Sex	−0.116	−0.239–0.007	0.065
**B. rs304**				
**Lipid**	**Variable**	**β-Coefficient**	**95% CI**	***p*** **-** **V** **alue**
TC	rs304	0.174	0.034–0.314	0.015
	Age	0.017	0.012–0.022	<0.001
	Sex (Female)	0.004	−0.136–0.143	0.959
LDL-C	rs304	0.174	0.050–0.298	0.006
	Age	0.010	0.006–0.014	<0.001
	Sex	−0.113	−0.237–0.010	0.071

TC: Total Cholesterol (mmol/L), LDL-C: Low-Density Lipoprotein–Cholesterol (mmol/L).

**Table 5:** Demographic data of Kuwaiti samples used in the sequencing and statistical analysis of the genetic variants of *LPL*.

Variables	Participants (*n* = 688)
Age (years)	32.92 ± 14.26
Sex (Female%)	414 (60.2%)
BMI (kg/m^2^)	27.17 ± 6.67
Cholesterol (mmol/L)	4.73 ± 0.94
Triglyceride (mmol/L)	1.06 ± 0.80
VLDL (mmol/L)	0.44 ± 0.33
HDL-C (mmol/L)	1.13 ± 0.32
LDL-C (mmol/L)	3.11 ± 0.81

## Data Availability

Raw genotypic data are available upon request for conducting meta-analysis.

## Supplementary Materials

The following supporting information can be downloaded at: https://www.mdpi.com/article/10.3390/ijms26157282/s1.

## Abbreviations

The following abbreviations are used in this manuscript:

SNP	Single-nucleotide Polymorphism
LPL	Lipoprotein Lipase
BMI	Body Mass Index
MAF	Minor Allele Frequency
HWE	Hardy–Weinberg Equilibrium
TG	Triglyceride
VLDL	Very-low-density Lipoprotein
HDL	High-density Lipoprotein
UTR	Untranslated Region
LD	Linkage Disequilibrium
