# Supplementary material for: Association of Lipoprotein Lipase (LPL) Variants rs8176337, rs303, and rs304 with Body Mass Index and Total Cholesterol
Source: Int J Mol Sci. 2025 Jul 28;26(15):7282. doi: 10.3390/ijms26157282 (PMC12346969; doi:10.3390/ijms26157282)
Supplement: Supplementary file 1 [file ijms-26-07282-s001.zip › ijms-3749230-supplementary.pdf]

Supplementary Material

Paper Title: Association of lipoprotein lipase (LPL) variants rs8176337, rs303, and rs304 with body mass index and total cholesterol

Authors: Ahmad Al-Serri; Suzanne A. Al-Bustan; Amani Al-Adsani, Sahar Barhoush; Lavina Miranda; Hala Hamdan; Babitha G. Annice; Majed A. Alnaqeeb

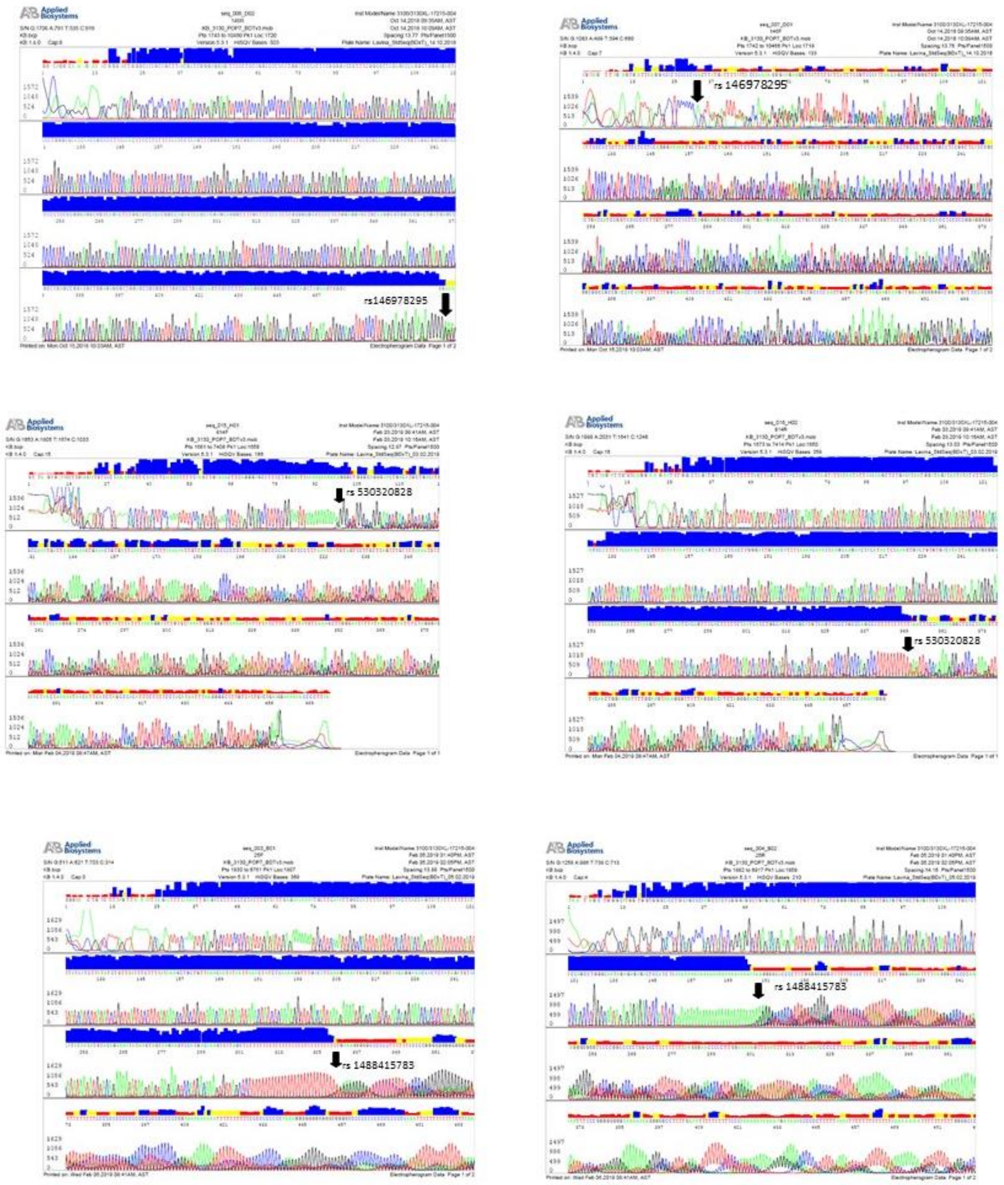

Supplementary Figure S1. Electropherogram of three target regions sequenced at the LPL gene locus generated by the reverse (A) primers showing the reported InDels. The peaks represent the various nucleotides detected which are discriminated by the fluorescent color. The blue box on top is the quality assurance (95%) of the base call.

Table S1. Identified variants in the targeted *LPL* regions and their characteristics.

Variant	Location	Position in the Gene	Type	Chromosome location	Global MAF	MAF N=715	Consequence (NCBI)	HWE p-value
rs80351041	5' UTR	5088 G>T	TRSV	8:19939158	0.0050 n=25	0.002	5'UTR variant	0.970
rs182193170	5' UTR	5308 C>A	TRSV	8:19939378	0.0004 n=2	0.001	5'UTR variant	0.985
rs146978295 (KUA LPL16)	Exon 1	5195-5196 Ins/CC	InDel	8:19939265	0.008 n=40	0.002	5'UTR variant	0.940
rs761167661	Exon 1	5441 G>C	TRSV	8:19939511	-	0.001	missense	0.985
rs75890454	Exon 1	5107 T>C	TRS	8:19939177	0.0038 n=19	0.002	5'UTR variant	0.970
rs1186635449	Exon 1	5420 G>A	TRS	8:19939490	-	0.001	missense	0.985
NOVEL SNP 1	Exon 1	5196 C>T	TRS	8:19939266		0.001	-	0.985
rs904972232	Intron 1	5563 G>A	TRS	8:19939633	-	0.001	Intron variant	0.985
rs1002894103	Exon 1	5094-5095 Ins/C	InDel	8:19939164	-	0.001	5'UTR variant	0.985
rs531586966	Intron 1	5670 A>G	TRS	8:19939740	0.0008 n=4	0.001	Intron variant	0.985
rs200412008	Intron 1	5488 C>T	TRS	8:19939558	0.0082 n=41	0.003	Intron variant	0.955
rs1800590	Exon 1	5090 T>G	TRSV	8:19939160	0.1340 n=671	0.057	5'UTR variant	0.836
rs141390463	Intron 1	5498 G>A	TRS	8:19939568	0.0124 n=62	0.004	Intron variant	0.925
rs796672773 (rs140116605)	Intron 2	14932-14933 del/TG	InDel	8:19949002	-	0.006	Intron variant	0.850
rs74377536	Intron 2	14987 C>A	TRSV	8:19949057	0.1180 n=591	0.139	Intron variant	0.140
rs7016529	Intron 2	15050 T>C	TRS	8:19949120	0.0871 n=436	0.042	Intron variant	**0.008**
rs8176337	Intron 2	15090 C>G	TRSV	8:19949160	0.3460 n=1733	0.204	Intron variant	0.433
rs114795370	Intron 2	14958 G>T	TRSV	8:19949028	0.0026 n=13	0.003	intron variant	0.940
rs530320828 (KUA LPL 26)	Intron 2	14875-14876 del/A	InDel	8:19948936	0.0407 n=204	0.050	Intron variant	**0.000**
KUA LPL-34	Intron 7	25465 T>A	TRSV	8:19959528	-	0.001	Intron variant	0.985
rs538674128 (KUA LPL-35)	Intron 7	25517 del/T	InDel	8:19959587	0.0010 n=5	0.005	Intron variant	0.880
rs303	Intron 7	25698 G>C	TRSV	8:19959768	0.1150 n=576	0.098	Intron variant	0.596
rs1488415783 (KUA LPL-36)	Intron 7	25708-25710 del/TTT	InDel	8:19959778	-	0.50	Intron variant	**0.000**

rs304	Intron 7	25780 T>G	TRSV	8:19959850	0.2532 n=1268	0.213	Intron variant	0.295
rs305	Intron 7	25820 A>G	TRS	8:19959890	0.2526 n=1265	0.212	Intron variant	0.273
rs306	Intron 7	25862 G>A	TRS	8:19959932	0.0206 n=103	A0.01	Intron variant	0.806
rs307	Intron 7	25874 A>G	TRS	8:19959944	0.0389 n=195	0.014	Intron variant	0.719
rs373767974	Intron 7	25812 G>A	TRS	8:19959882	0.0004 n=2	0.002	Intron variant	0.970
rs188533806	Intron 7	25811 C>T	TRS	8:19959881	0.0010 n=5	0.005	Intron variant	0.895
rs181485825	Intron 7	25833 C>A	TRSV	8:19959903	0.0012 n=6	0.005	Intron variant	0.895

TRSV: Transversion, TRS: Transition, MAF: Minor Allele Frequency, HWE: Hardy-Weinberg Equilibrium, KU LPL-#: Reported as novels [18]. The shaded SNPs were selected for genetic association in this study.

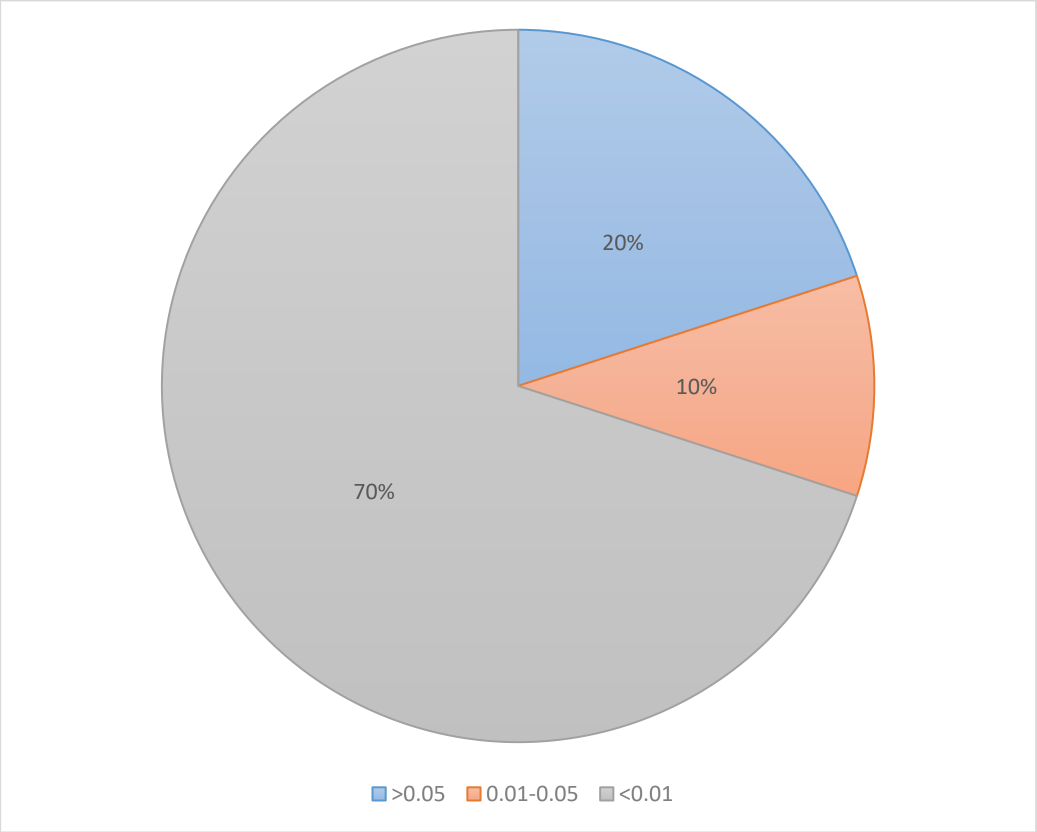

Supplementary Figure S2. The distribution of the variants identified based on their Minor Allele Frequency (MAF).

Supplementary Table S2. Genotype distribution of all the variants identified among the original samples sequenced.

Genetic Variants	Genotypes and Alleles	Total	Genetic Variants	Genotypes and Alleles	Total
		**n=715**			**n=715**
**rs200412008**	C/C	0.996 (n=712)	**rs75890454**	T/T	0.997 (n=713)
	C/T	0.004 (n=3)		T/C	0.003 (n=2)
	T/T	0.000 (n=0)		C/C	0.000 (n=0)
	C	0.998		T	0.999
	T	0.002		C	0.001
	**HWE (p)**	0.955		**HWE (p)**	0.970
**rs182193170**	C/C	0.999 (n=714)	**rs1186635449**	G/G	0.999 (n=714)
	C/A	0.001 (n=1)		G/A	0.001 (n=1)
	A/A	0.000 (n=0)		A/A	0.000 (n=0)
	C	0.999		A	0.999
	A	0.001		G	0.001
	**HWE (p)**	0.985		**HWE (p)**	0.985
**rs761167661**	G/G	0.999 (n=714)	**rs80351041**	G/G	0.997 (n=713)
	G/C	0.001 (n=1)		G/T	0.003 (n=2)
	C/C	0.000 (n=0)		T/T	0.00 (n=0)
	G	0.999		G	0.999
	C	0.001		T	0.001
	**HWE (p)**	0.985		**HWE (p)**	0.970
**rs1800590**	T/T	0.890 (n=636)	**NOVEL SNP 1**	C/C	0.999 (n=714)
	T/G	0.108 (n=77)		C/T	0.001 (n=1)
	G/G	0.003 (n=2)		T/T	0.000 (n=0)
	T	0.943		C	0.999
	G	0.057		T	0.001
	**HWE (p)**	0.837		**HWE (p)**	0.985
**rs146978295**	-	0.994 (n=711)	**rs74377536**	C/C	0.736 (n=526)
	-/CC	0.006 (n=4)		C/A	0.252 (n=180)
	CC	0.000 (n=0)		A/A	0.013 (n=9)

	-	0.997		C	0.862
	CC	0.003		A	0.138
	**HWE (p)**	0.940		**HWE (p)**	0.140
**rs904972232**	G/G	0.999 (n=714)	**rs8176337**	C/C	0.639 (n=457)
	G/A	0.001 (n=1)		C/G	0.315 (n=225)
	A/A	0.000 (n=0)		G/G	0.046 (n=33)
	G	0.999		C	0.797
	A	0.001		G	0.203
	**HWE (p)**	0.985		**HWE (p)**	0.434
**rs141390463**	G/G	0.993 (n=710)	**rs796672773**	TG	0.000 (n=0)
	G/A	0.007 (n=5)		TG/-	0.014 (n=10)
	A/A	0.000 (n=0)		-	0.986 (n=705)
	G	0.997		TG	0.007
	A	0.003		-	0.993
	**HWE (p)**	0.925		**HWE (p)**	0.851
**rs1002894103**	-	0.999 (n=714)	**rs530320828**	A	0.000 (n=0)
	-/C	0.001 (n=1)		A/-	1.000 (n=715)
	C	0.000 (n=0)		-	0.000 (n=0)
	-	0.999		A	0.500
	C	0.001		-	0.500
	**HWE (p)**	0.985		**HWE (p)**	**0.000**
**rs531586966**	A/A	0.999 (n=714)	**rs7016529**	T/T	0.923 (n=660)
	A/G	0.001 (n=1)		T/C	0.071 (n=51)
	G/G	0.000 (n=0)		C/C	0.006 (n=4)
	A	0.999		T	0.959
	G	0.001		C	0.041
	**HWE (p)**	0.985		**HWE (p)**	**0.009**
**rs114795370**	**G/G**	0.994 (n=711)	**rs306**	G/G	0.982 (n=702)
	G/T	0.006 (n=4)		G/A	0.018 (n=13)
	T/T	0.000 (n=0)		A/A	0.000 (n=0)

	G	0.997		G	0.991
	T	0.003		A	0.009
	**HWE (p)**	0.940		**HWE (p)**	0.806
**rs303**	G/C	0.817 (n=584)	**rs188533806**	C/C	0.990 (n=708)
	G/C	0.172 (n=123)		C/T	0.010 (n=7)
	C/C	0.011 (n=8)		T/T	0.000 (n=0)
	G	0.903		C	0.995
	C	0.097		T	0.005
	**HWE (p)**	0.596		**HWE (p)**	0.895
**rs304**	T/T	0.627 (n=448)	**rs181485825**	C/C	0.990 (n=708)
	T/G	0.322 (n=230)		C/A	0.010 (n=7)
	G/G	0.052 (n=37)		A/A	0.000 (n=0)
	T	0.787		C	0.995
	G	0.213		A	0.005
	**HWE (p)**	0.295		**HWE (P)**	0.895
**rs305**	A/A	0.627 (n=448)	**rs538674128**	T	0.000 (n=0)
	A/G	0.322 (n=230)		T/-	0.011 (n=8)
	G/G	0.052 (n=37)		-	0.989 (n=707)
	A	0.787		T	0.006
	G	0.213		-	0.994
	**HWE (p)**	0.295		**HWE (p)**	0.880
**rs1488415783**	TTT	0.000 (n=0)	**rs307**	A/A	0.973 (n=696)
	TTT/-	1.000 (n=715)		A/G	0.027 (n=19)
	-	0.000 (n=0)		G/G	0.000 (n=0)
	TTT	0.500		A	0.987
	-	0.500		G	0.013
	**HWE (p)**	**0.000**		**HWE (p)**	0.719
**rs373767974**	G/G	0.997 (n=713)	KUA LPL-34	T/T	0.999 (n=714)
	G/A	0.003 (n=2)		T/A	0.001 (n=1)
	A/A	0.000 (n=0)		A/A	0.000 (n=0)
	G	0.999		T	0.999
	A	0.000		A	0.001
	**HWE (p)**	0.970		**HWE (p)**	0.985

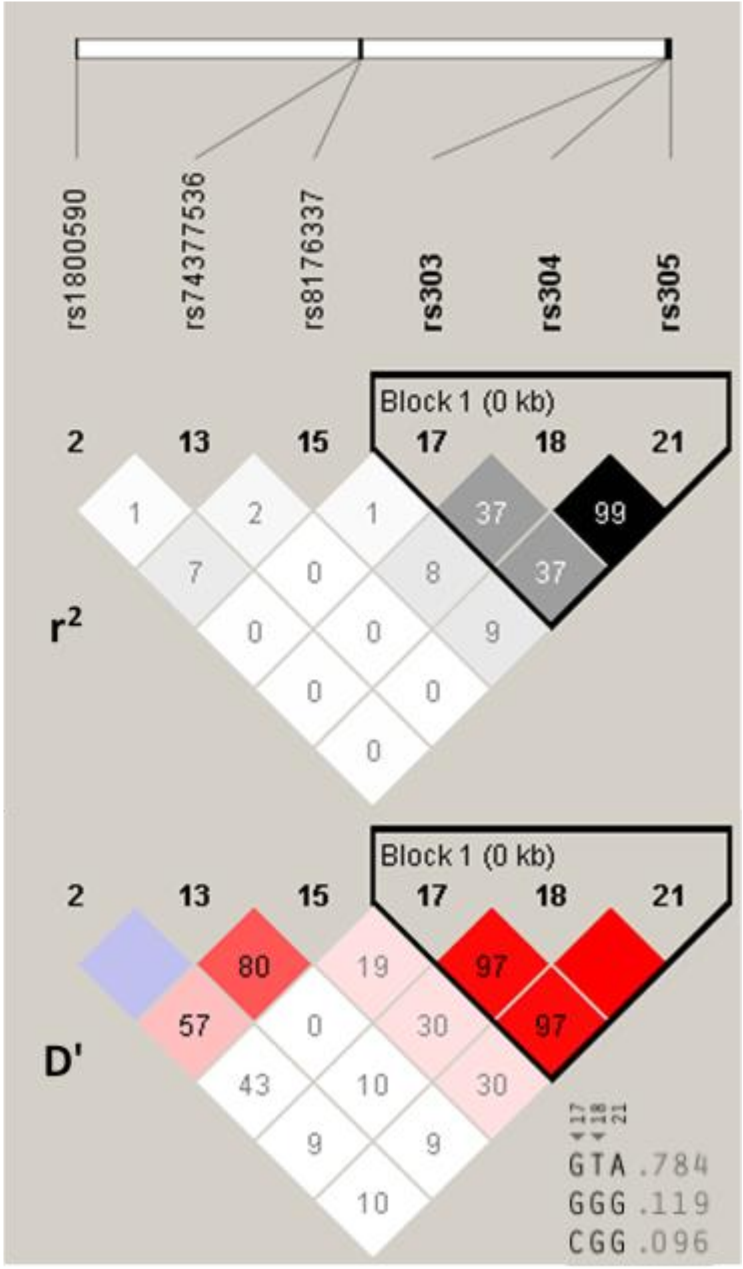

Supplementary Figure S3. Linkage disequilibrium (LD) pattern and haplotype of the six common *LPL* SNPs in the Kuwaiti population. One haplotype block was created consisting of three haplotypes shown at the bottom right. Numbers inside each pair indicate r^2 or D' values expressed as percentile. Strong LD is indicated by black or dark grey color of r^2 and bright red color of D'/LOD.

Supplementary Table S3. Genotype distribution and minor allele frequency (MAF) for the five selected SNPs in 688 Kuwaiti samples. The significance of HWE result was indicated by *p*-value where CI is 95%.

SNP	W/W	W/M	M/M	HWE *p*-value	MAF
**rs1800590 T>G**	610	76	2	0.821	G: 0.058
**rs74377536 C>A**	503	176	9	0.141	A: 0.140
**rs8176337 C>G**	437	218	33	0.390	G: 0.206
**rs303 G>C**	562	118	8	0.522	C: 0.097
**rs304 T>G**	428	224	36	0.347	G: 0.215

-W/W indicates the major (wild) homozygous genotype
-W/M indicates the heterozygous genotype
-M/M indicates the minor (mutant) homozygous genotype

Supplementary Table S4: Functional prediction identified by the Human Splice Finder tool for the investigated *LPL* variants. (Desmet et al, 2009)

SNP	Functional analysis
**rs1800590** **(**ENST00000311322.8:c.-281T>G)	Tool cannot analyze the SNP
**rs74377536** **(**ENST00000311322.8: **c.249+717C>A)**	Creation of an intronic (exon splicing enhancer) ESE site.<br>Probably no impact on splicing.
**rs8176337** **(**ENST00000311322.8: c.249+820C>G)	Activation of an intronic cryptic donor site.<br>Potential alteration of splicing.
**rs303** (ENST00000311322.8: c.1139+388G>C)	Alteration of an intronic ESS site (exon splicing silencer).<br>Probably no impact on splicing.
**rs304** **(**ENST00000311322.8: c.1139+470T>G)	No significant splicing motif alteration detected. This mutation has probably no impact on splicing<br>.402
